# Enhanced oral delivery of hesperidin-loaded sulfobutylether-β-cyclodextrin/chitosan nanoparticles for augmenting its hypoglycemic activity: in vitro-in vivo assessment study

**DOI:** 10.1007/s13346-023-01440-6

**Published:** 2023-10-16

**Authors:** Mona Ebrahim Elmoghayer, Noha Mohamed Saleh, Irhan Ibrahim Abu Hashim

**Affiliations:** Department of Pharmaceutics, Faculty of Pharmacy, Mansoura University, Mansoura, 35516 Egypt

**Keywords:** Hesperidin, Hypoglycemic, Sulfobutylether-β-cyclodextrin, Chitosan, Nanoparticles, Release, Permeation

## Abstract

Hesperidin (Hsd), a bioactive phytomedicine, experienced an antidiabetic activity versus both Type 1 and Type 2 *Diabetes mellitus*. However, its intrinsic poor solubility and bioavailability is a key challenging obstacle reflecting its oral delivery. From such perspective, the purpose of the current study was to prepare and evaluate Hsd-loaded sulfobutylether-β-cyclodextrin/chitosan nanoparticles (Hsd/CD/CS NPs) for improving the hypoglycemic activity of the orally administered Hsd. Hsd was first complexed with sulfobutylether-β-cyclodextrin (SBE-β-CD) and the complex (CX) was found to be formed with percent complexation efficiency and percent process efficiency of 50.53 ± 1.46 and 84.52 ± 3.16%, respectively. Also, solid state characterization of the complex ensured the inclusion of Hsd inside the cavity of SBE-β-CD. Then, Hsd/CD/CS NPs were prepared using the ionic gelation technique. The prepared NPs were fully characterized to select the most promising one (F1) with a homogenous particle size of 455.7 ± 9.04 nm, a positive zeta potential of + 32.28 ± 1.12 mV, and an entrapment efficiency of 77.46 ± 0.39%. The optimal formula (F1) was subjected to further investigation of in vitro release, ex vivo intestinal permeation, stability, cytotoxicity, and in vivo hypoglycemic activity. The results of the release and permeation studies of F1 manifested a modulated pattern between Hsd and CX. The preferential stability of F1 was observed at 4 ± 1 °C. Also, the biocompatibility of F1 with oral epithelial cell line (OEC) was retained up to a concentration of 100 µg/mL. After oral administration of F1, a noteworthy synergistic hypoglycemic effect was recorded with decreased blood glucose level until the end of the experiment. In conclusion, Hsd/CD/CS NPs could be regarded as a hopeful oral delivery system of Hsd with enhanced antidiabetic activity.

**Graphical Abstract:**

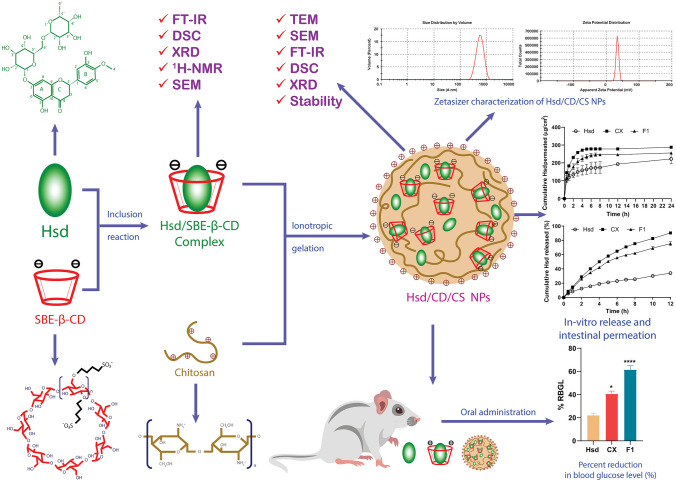

## Introduction

**Fig. 1:**
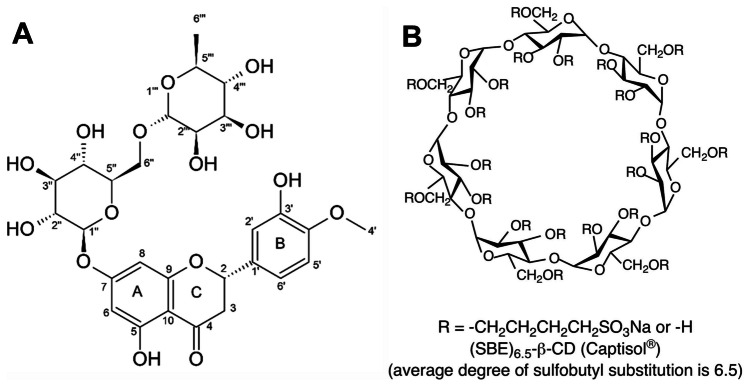
Chemical structures of hesperidin (**A**) [2] and sulfobutylether-ß-cyclodextrin (**B**) [162]

Hesperidin (Hsd) (30,5,7-trihydroxy 40-methoxy flavanone 7-rutinoside) is a phytomedicine that is found plentifully in citrus fruits, such as oranges, mandarins, lemons, limes, and grapefruits (Fig. 1A) [1, 2]. It is a fundamental flavanone glycoside in the peel of sweet oranges [2–4]. Hsd has been identified as a promising phytoconstituent with antibacterial, hypolipidemic, antiallergic, antihypertensive, cardioprotective, and anticancer properties [5–7]. Additionally, it has shown a potent antioxidant activity which helps to safeguard against various chronic diseases such as *Diabetes mellitus* [2, 8]. Hsd depicts auspicious antidiabetic action as it improves insulin sensitivity, and therefore prevents hyperglycemia in both Type 1 and Type 2 diabetes [9–11]. It was also reported that Hsd had protective effects against diabetic nephropathy and neuropathy [12, 13]. Despite the hopeful therapeutic outcomes of Hsd, its poor aqueous solubility, and bioavailability have limited its oral delivery [14, 15]. Thus, an approach that could enhance the solubility of Hsd such as complexation might be helpful to improve its dissolution, bioavailability, stability, and biological activity [1]. Also, the loading of Hsd in bioactive polymer-based (NPs) would provide an innovative delivery system for further enrichment of the in vivo bioactivity.

Beta-cyclodextrin (β-CD) is a cyclic oligosaccharide consisting of seven d-glucopyranose units connected by 1,4-glucosidic bonds [16–18]. β-CDs have a central hydrophobic cavity and outer hydrophilic surface, enabling them to encapsulate lipophilic drugs [19–21]. A modification of β-CDs via the addition of sulfobutyl moiety to form sulfobutylether-β-CD (SBE-β-CD, Fig. 1B) shows greatly enhanced aqueous solubility, permeability, bioavailability, biocompatibility, low toxicity, and amended complexing ability for lipophilic drugs compared to the parent β-CD [22–24].

Chitosan (CS) is a natural bioactive linear polysaccharide with N-acetyl glucosamine units linked with glucosamine ones by β-1,4-glucosidic bonds [25–27]. CS demonstrated versatile pharmacological activities such as anti-diabetic, antioxidant, anti-allergic, anti-inflammatory, anticoagulant, antibacterial, and antiviral activities [28, 29]. The ionic gelation of CS with polyanions as SBE-β-CD is one of the most popular and simple approaches for the formulation of NPs. The negatively charged sulfonate groups of SBE-β-CD crosslink with the positively charged amino groups of CS in an environment-friendly non-organic aqueous medium [30, 31]. CS has a unique feature of bearing a positive charge unlike other polysaccharides, allowing it to interact and bind strongly with negatively charged biological surfaces [32, 33]. Moreover, CS NPs improve drug penetration, safeguard acid-sensitive medicines, boost drug release in basic pH, and offer excellent oral bioavailability [34, 35]. Interestingly, CS-based NPs are promising carriers for the delivery and complementation of antidiabetic drugs [36].

To the best of our knowledge, limited trials have been conducted to increase the solubility of Hsd via complexation. At the same time, few studies reported the heightened antidiabetic activity of Hsd NPs [37–39]. Hence, this study is the first one aimed to enhance the solubility of Hsd by its complexation with SBE-β-CD. Then, our effort focused on the development of CS-based NPs via the ionic gelation technique to serve as an oral delivery system of Hsd for augmenting its hypoglycemic activity.

## Materials and methods

### Materials

CS (Mwt 50-190 kDa, deacetylation percent 85%) and porcine stomach mucin were purchased from Sigma-Aldrich (Saint Louis, MO, USA). Hsd was obtained from Abcam (Cambridge, MA, USA). SBE-β-CD sodium salt; Captisol^®^ (Mwt 2160) was kindly supplied by Cydex L.C. (USA). Millipore filters were obtained from Millipore Corporation (Bedford, MA 01730, USA). Oral epithelial cell (OEC) was gotten from Nawah Scientific Inc., (Mokatam, Cairo, Egypt). Glucose TR oxidase peroxidase kit (GOD/POD) was bought from Spinreact, S.A.U., Spain. Other chemicals were of analytical grades and were used without additional treatment.

### Methodology

#### Phase solubility

An excess amount of Hsd was added to SBE-β-CD aqueous solutions (0.0–3 mM) in 10-mL screw-capped tubes. The tubes were placed in a thermostatic shaker bath for 24 h at 25 ± 0.5 and 37 ± 0.5 °C (Grant Instrument, Cambridge Ltd., UK) [40–42]. Then, the tubes were centrifuged for 1 h at 10,000 rpm (Centrifuge, Hettich Micro 22 R, Germany). An aliquot (3 mL) of the supernatant was filtered (0.45 µm) and measured spectrophotometrically at 284 nm after proper dilution (UV/VIS spectrophotometer, JASCO, Tokyo, Japan). The phase solubility curve of Hsd (mM) versus SBE-β-CD (mM) was constructed and the slope was calculated. The stability constant (*K*_1:1_) of the Hsd/SBE-β-CD inclusion complex (CX) was determined as follows [43, 44]:

1 $${\mathrm{K}}_{1:1}= \frac{\mathrm{Slope}}{{S}_{0}(1-\mathrm{Slope})}$$

where *S*_0_ is the intrinsic solubility of Hsd in the absence of SBE-β-CD.

#### Preparation of Hsd/SBE-β-CD complex (CX) and physical mixture

The inclusion complex of SBE-β-CD with Hsd was prepared by the freeze-drying technique with some modifications [45]. First, SBE-β-CD (54.1 mg) was dissolved in 25 mL distilled water. Hsd (15.3 mg) was dissolved in 18 mL methanol using water bath sonication for 10 min (Sonix IV, model ss101H230, USA). Then, the aqueous solution of SBE-β-CD was dropped quickly onto the Hsd solution under magnetic stirring (Misung Scientific Co. model MS-300HS, Ltd, Korea). The obtained mixture was shaken for 48 h at 37 °C (GFL Gesellschaft für Labortechnik, Burgwedel, Germany). After that, the clear mixture was freeze-dried for 24 h (Freeze dryer, SIM FD8-8T, SIM International, USA), and the dried CX was collected, weighed, and kept for further evaluation. A physical mixture of Hsd and SBE-β-CD was prepared in a 1:1 molar ratio by gently mixing the two components [46].

#### Characterization of Hsd/SBE-β-CD complex (CX)

##### Percent complexation and process efficiency

The freeze-dried CX (5 mg) was solubilized in 2.5 mL distilled water (to dissolve the complexed Hsd only) and another 5 mg of the CX was dissolved in 2.5 mL methanol (to dissolve total Hsd (both free and complexed Hsd)). Then, both solutions were sonicated in a water bath sonicator for 10 min. The obtained solutions were filtered to remove the undissolved Hsd. After proper dilution, the Hsd concentration in water (complexed Hsd) and methanol (total Hsd (free and complexed)) was measured spectrophotometrically at 284 nm [47]. Also, the yield of the CX or process efficiency was stated as the percentage of the mass of the recovered CX (lyophilized powder) over the sum mass of the initial components (Hsd + SBE-β-CD). The percent complexation efficiency (%CE) and the percent process efficiency (%PE) were calculated as follows [48, 49]:

2 $$\%\mathrm{CE}= \frac{\text{Complexed Hsd}}{\text{Total Hsd}} \times 100$$

3 $$\%\text{PE}= \frac{\text{wt of Cx}}{\text{wt of Hsd}\mathop{+}\text{wt of SBE} - \upbeta - \mathrm{CD}}\times 100$$

##### Fourier transform-infrared (FT-IR) spectroscopy

The FT-IR spectra of Hsd, SBE-β-CD, their binary physical mixture (Ph_CX_) with the same ratios of Hsd and SBE-β-CD in CX, and CX were determined using FT-IR spectrophotometer (Madison Instruments, Middleton, WI, USA). The analysis was conducted using the KBr technique. Individual samples (2 mg each) were milled with KBr (200 mg), compressed, and analyzed over a range of 4000–500 cm^−1^.

##### Differential scanning calorimetry (DSC)

Thermograms of Hsd, SBE-β-CD, Ph_CX_, and CX were obtained utilizing the DSC technique (DSC 6000; Perkin-Elmer, Waltham, MA, USA). The samples were weighed (4 mg each) and heated gradually in aluminum pans under a dry nitrogen stream purging at 20 mL/min. The temperature range and the heating rate were 50–350 °C and 10 °C/min, respectively. For the temperature calibration, indium was applied as a reference standard during DSC runs.

##### X-ray diffractometry (XRD)

X-ray diffraction patterns of Hsd, SBE-β-CD, Ph_CX_, and CX were recorded using an X-ray diffractometer equipped with Cu Kα (Diano Corp., Woburn, MA, USA). The examination was conducted over a scanning range from 3 to 50° at 2*θ* angle at a current and a voltage of 9 mA and 45 kV, respectively.

##### Proton-nuclear magnetic resonance (^1^H-NMR)

^1^H-NMR spectra were obtained using a Bruker DRX spectrometer (Bruker Daltonics Inc., MA, USA) at 600 MHz and JEOL ECA 500 II spectrometers (Japan) at 500 MHz, and Bruker Ascend^™^ 400 spectrometer (Bruker Daltonics, Bremen, Germany) at 400 MHz. The applied solvent was DMSO-*d*_*6*._ Hsd was accurately weighed (1 mg) and dissolved in 500 µL DMSO, while CX (1 mg) was dissolved in 1 mL DMSO. The obtained data were processed using MestreNova processor software. Chemical shifts were measured in *ppm* on the δ scale regarding the TMS resonance; coupling constants (*J*) were expressed in Hz.

##### Scanning electron microscopy (SEM)

The topographical characteristics of Hsd, SBE-β-CD, Ph_CX_, and CX were explored using SEM worked at an acceleration voltage of 20 kV (JSM 6150, JEOL, Tokyo, Japan). Individual samples were loaded on stubs. Then, the fixed samples were covered with a gold/palladium alloy with the JFC1200 Fine Coater (JEOL, Tokyo, Japan). The pre-prepared samples were examined and microphotographs were acquired at a suitable magnification.

#### Preparation of hesperidin/sulfobutylether-β-cyclodextrin/chitosan nanoparticles (Hsd/CD/CS NPs)

As reported, it was essential to perform preliminary trials of blank NPs preparation by ionotropic gelation method to detect NPs formation zone as a prerequisite step for medicated NPs fabrication with optimum characteristics [50, 51]. Thus, solutions of SBE-β-CD and CS at a concentration range of 0.25 to 2% w/v were prepared. SBE-β-CD and CS were dissolved in distilled water and acetic acid aqueous solution (1% v/v), respectively. The SBE-β-CD solution was transferred into a syringe and added in a dropwise manner to the CS solution (20 mL) at room temperature till the clear solution turned opaque (formation of NPs) [52]. This phase transition was identified as the formation of NPs. Based on the obtained finding, CS at the concentration of 0.5% w/v (5 mg/mL) was chosen for the preparation of all the coming NPs due to its ease of handling, suitable viscosity, and lack of clumping.

In this study, the formulation of Hsd/CD/CS NPs passed through two steps. The first one was to prepare CX by the above-mentioned method. Then, the CX was dissolved in deionized water (DIW) at a concentration of 10 mg/mL. The CX solution was gradually dropped into 10 mL of CS solution (5 mg/mL) till the clear solution turned opaque [53]. The NPs were separated by cooling centrifugation (Acculab CE16-4X100RD, USA) at 13,000 rpm for 2 h. The obtained cakes were collected, freeze-dried, and kept for further evaluation. In the same manner, plain CD/CS NPs were prepared but by using a pure SBE-β-CD solution instead of the CX one. The constituents of the prepared NPs are listed in Table 1.

**Table 1:** The compositions of Hsd/CD/CS nanoparticles

**Formula**	**CS:CX**	**CS (mg)**	**SBE-β-CD equivalent amount (mg) in CX**
**F1**	1:1.5	50	75 mg
**F2**	1:1.25	50	62.5 mg
**F3**	1:1	50	50 mg
**F4**	1:0.8	50	40 mg
**F5**	1:0.67	50	33.5 mg

*CS* chitosan *SBE-β-CD* sulfobutylether-β-cyclodextrin, *CX* Hsd/SBE-β-CD complex

#### Characterization of Hsd/CD/CS NPs

##### Hsd entrapment efficiency

The entrapment efficiency percentage (EE%) was assessed indirectly. The unentrapped Hsd (free Hsd) in Hsd/CD/CS NPs dispersion was separated by cooling centrifugation at 13,000 rpm for 2 h. Then, the supernatant was quantified spectrophotometrically at 284 nm versus the corresponding supernatant of the plain CD/CS NPs as a blank after proper dilution. The EE% was calculated as follows [54, 55]:

4 $$\mathrm{EE\%}= \frac{{\mathrm{Hsd}}_{\mathrm{t}}-{\mathrm{Hsd}}_{\mathrm{f}}}{{\mathrm{Hsd}}_{\mathrm{t}}}\times 100$$

where Hsd_t_ is the total of Hsd and Hsd_f_ is the unentrapped Hsd in the supernatant.

##### Particle size, polydispersity index, and zeta potential

After proper dilution with DIW, the particle size, polydispersity index (PDI), and zeta potential (ZP) of Hsd/CD/CS NPs were measured using Malvern Zetasizer Nano ZS (Malvern Instruments Limited, UK). Dynamic Light Scattering (DLS) and Laser Doppler Micro-Electrophoresis procedures were employed for particle size and ZP measurements, respectively.

##### Mucoadhesive strength

The assessment of the mucoadhesive strength of Hsd/CD/CS NPs was related to the electrostatic attraction between mucin (negatively charged) and NPs (positively charged) [56]. Equal volumes of NPs dispersion and mucin solution (0.5 mg/mL) in phosphate buffer saline (pH 7.4) were magnetically stirred for 1 h at 37 °C. Then, the obtained mixture was centrifuged at 11,000 rpm for 1 h (Benchtop Centrifuge, Sigma Laborzentrifugen GmbH, Germany). The total mucin (before interaction with the NPs) and the free one (in the supernatant) were measured spectrophotometrically at 251 nm [32, 57, 58]. The mucoadhesive strength of Hsd/CD/CS NPs expressed as mucin binding efficiency (%) was calculated as follows:

5 $$\text{Mucin binding efficiency }(\mathrm{\%})=\frac{{\mathrm{Mucin}}_{\mathrm{t}}- {\mathrm{Mucin}}_{\mathrm{f}}}{{\mathrm{Mucin}}_{\mathrm{t}}}\times 100$$

where Mucin_t_ is the total mucin and Mucin_f_ is the free mucin in the supernatant.

Based on the characterization of Hsd/CD/CS NPs, an optimized formula (F1) was selected to undergo further investigations.

#### Characterization of the optimal formula (F1)

##### Transmission electron microscopy (TEM)

The morphological characteristics of F1 were observed by TEM (JEOL 2100; JEOL, Tokyo, Japan). A drop of a fresh F1 dispersion was spread over a carbon-coated copper grid and then extra dispersion was removed by a filter paper. The sample was allowed to dry at room temperature and directly inspected without staining. The microphotographs were captured at proper magnification.

##### SEM

The topography of CX, CS, physical mixture of CX and CS (Ph_NPs_), and F1 was investigated using SEM operated at an acceleration voltage of 20–30 kV. The sample preparation was conducted as mentioned before under the “Scanning electron microscopy (SEM)” section.

##### Solid state characterization

The FT-IR spectra, DSC thermograms, and X-ray diffraction patterns of CX, CS, and Ph_NPs_ as well as the freeze-dried samples of F1 and the corresponding plain NPs (PLF1) were measured as mentioned under the “Characterization of Hsd/SBE-β-CD complex (CX)” section.

#### In vitro release

The in vitro release of Hsd, CX, and F1 was studied using modified vertical Franz diffusion cells with a diffusional surface area of 5.12 cm^2^/cell. A dialysis cellulose membrane (Mwt cut off 12,000–14,000 Da, Sigma-Aldrich, Saint Louis, MO, USA) was pre-equilibrated overnight with the release media. Then, donor and receptor half-cells were separated using the cellulose membrane. The methanolic buffered solutions at pH 1.2, 6.8, and 7.4 were applied as release media to simulate the gastric, intestinal, and physiological pH, respectively. Methanol was added at a concentration of (40% v/v) to secure sink conditions throughout the experiment [59]. Briefly, aqueous CX solution, F1 dispersion, and aqueous Hsd dispersion equivalent to 0.7 mg Hsd in DIW were introduced into the donor compartments. The release media (100 mL/cell) were placed in the receptor ones. The Franz cells were placed in a thermostatically controlled incubator kept at 37 ± 0.5 °C and shaken at 100 rpm throughout the experiment (GFL Gesellschaft für Labortechnik, Burgwedel, Germany). During the experiment, samples were withdrawn at predetermined time points and replaced with an equal volume of fresh medium equilibrated at the same conditions. The gathered samples were analyzed spectrophotometrically at 284 nm. Ultimately, the cumulative Hsd released (%) was determined at each time point and plotted versus the corresponding time.

#### Kinetic analysis

To acquire a comprehensive understanding of the Hsd release mechanism_,_ the release data were fitted to kinetic models that were zero-order (*A* vs. *t*), first-order (log $\left(A_{\mathrm o}-A\right)$ vs. *t*), Higuchi (*A* vs. *t*^1/2^) [60], and Korsmeyer-Peppas equation $\left(A_{\mathrm t}/A_\infty={k_pt}^n\right)$ [61]. Where, *A* is the cumulative percent Hsd released at time t, $\left(A_{\mathrm o}-A\right)$ is the percent of the remaining Hsd after time *t*, $A_{\mathrm t}/A_\infty$ is the fraction of Hsd released after time *t*, *k*_p_ is the Korsmeyer-Peppas kinetic constant, and *n* is the release diffusional exponent (*n* = slope of log $A_{\mathrm t}/A_\infty$ vs. log *t*). The kinetic model having the highest coefficient of determination (*R*^2^) was stated as the Hsd release mechanism [62, 63].

#### Ex vivo intestinal permeation

An excised goat small intestine was obtained from the local slaughterhouse. The sections of intestinal tissue were freshly isolated, dissected longitudinally, and washed in a series of saline solutions under sterile conditions. Then, the sample tissues were kept in saline at −20 °C until experimenting. The Research Ethical Committee at the Faculty of Pharmacy, Mansoura University permitted protocols involving the excision of the tissue samples and the animal experiments (Ethical Approval Code: 2023-129) following “The Principle of Laboratory Animal Care” (NIH publication No. 85-23, revised 1985). Also, the animal-related experiments were completed in agreement with the Animal Research: Reporting of In Vivo Experiments (ARRIVE) principles.

The ex vivo permeation study was conducted using the modified vertical Franz cell with a diffusional area of 5.12 cm^2^/cell. The intestine samples were positioned between the donor and receptor compartments with the inner mucous side facing the donor compartment. The methanolic buffered solution (40% v/v) at pH 7.4 was employed as a permeation medium. Then, the permeation experiment was conducted using the same condition stated under the “In vitro release” section. with Hsd amount equivalent to 0.7 mg in DIW (CX solution, F1 dispersion, and Hsd dispersion) was introduced into the donor compartments. The amount of Hsd permeated across the intestine per unit surface area (dM/*A*) (µg/cm^2^) was plotted versus time. After that, the slope of the linear part of the constructed permeation curve was designated as the steady-state flux (*J*_ss_, µg/cm^2^.h). The permeability coefficient (*K*_p_, cm/h) was estimated by dividing the *J*_ss_ over the donor concentration of Hsd (*C*_0_, µg/mL) [64–66].

#### Stability study

The stability of F1 was studied at refrigerator (4 ± 1 °C) and ambient temperature throughout 6 months [67–69]. Freshly prepared F1 dispersions were stored in amber glass screw-capped bottles at the above-mentioned temperatures. The particle size, PDI, ZP, and drug retention percentage (DR%) of NPs were assessed at zero, 1 month, 3 months, and 6 months. The DR% was calculated as follows [70]:

6 $$\text{Drug retention }(\mathrm{DR\%})=\frac{\mathrm{EE\% \;at \;each \;time \;interval}}{\mathrm{EE\% \;at\; zero \;time}}\times 100$$

where EE% is the Hsd entrapment efficiency percentage inside NPs.

#### Cytotoxicity assay

Cytotoxicity assay was conducted using a normal oral epithelial cell line (OEC). The water-soluble tetrazolium salt I (WST-1) technique was applied. OEC was preserved in DMEM media enriched with 100 units/mL of penicillin, 100 mg/mL of streptomycin, and 10% of heat-inactivated fetal bovine serum in a humidified atmosphere of 5% (v/v) CO_2_ at 37 °C. Aliquots of cell suspension (50 µL, 3 × 10^3^ cells) were cast in 96-well plates and incubated for 24 h in complete media. Then, additional aliquots of 50 µL media containing Hsd, CX, PLF1, and F1 at concentrations of 0.01, 0.1, 1, 10, and 100 µg/mL were added to the cells. The treated cells were incubated for 48 h. After that, cells were further treated with WST-1 (10 µL/well), and the absorbance was measured spectrophotometrically after 1 h at 450 nm using a microplate reader (Allmendgrün, Ortenberg). Percent cell viability (%) was estimated using the absorbance of treated cells and that of the negative control cells. Then, the inhibitory concentration of 50% of cells (IC_50_) was calculated by plotting percent cell viability versus log concentrations [71, 72].

7 $$\text{Cell viability }(\mathrm{\%})=\frac{\mathrm{ A}450\text{ of treated cells }}{\mathrm{A}450\text{ of controlled cells}}\times 100$$

#### In vivo evaluation of the pharmacodynamic hypoglycemic effect

Fifteen male Sprague-Dawley rats (ca 190–200 g) were used in this study. The Research Ethical Committee at the Faculty of Pharmacy, Mansoura University approved protocols involving the excision of the tissue samples and the animal experiments (Ethical Approval Code: 2023-129) under “The Principle of Laboratory Animal Care” (NIH publication No. 85-23, revised 1985). Also, the animal experiments were performed in agreement with the Animal Research: Reporting of In Vivo Experiments (ARRIVE) principles. Two weeks before the experiment, the animals were adapted to an environmentally monitored room with free access to a standard diet and water ad libitum. Before the experiment, the animals were fasted for 12 h with water ad libitum. At random, the animals were partitioned into three groups (five animals per group):

- Group I: Oral Hsd administration.
- Group II: Oral administration of Hsd/SBE-β-CD complex (CX).
- Group III: Oral administration of Hsd/CD/CS NPs (F1).

Each animal served as its control by determining the blood glucose level in the blood sample that was withdrawn before the oral administration (BG_0_). The animals of Group I, II, and III received oral doses of Hsd aqueous dispersion, CX aqueous solution, and F1 dispersion, respectively, equivalent to 50 mg Hsd/kg. Blood samples were collected from the retro-orbital venous plexus at 0, 1, 2, 4, 6, 9, and 12 h after the oral administration [73, 74]. The glucose oxidase/peroxidase method was employed to estimate the blood glucose level (BG_t_) using the GOD/POD kit. The percentage reduction in the blood glucose level (%RBGL) was possessed as a reversal for the hypoglycemic response that was calculated as follows [75, 76]:

8 $$\mathrm{\%RBGL}=\frac{{\mathrm{BG}}_{0}-{\mathrm{BG}}_{\mathrm{t}}}{{\mathrm{BG}}_{0}}\times 100$$

where BG_0_ is the blood glucose at zero time and BG_t_ is the blood glucose at a certain time interval.

#### Statistical analysis

In vitro and in vivo data of this study were stated as mean ± standard deviation (SD) and mean ± standard error of the mean (SEM), respectively. Statistical analysis was conducted by one-way analysis of variance (ANOVA) followed by a multiple comparisons test (Tukey-Kramer) using GraphPad Prism software version 8.00 (San Diego, CA, USA). The *P* values at level *P* < 0.05 were considered statistically significant.

## Results and discussion

### Characterization of Hsd/SBE-β-CD complex (CX)

#### Phase solubility

**Fig. 2:**
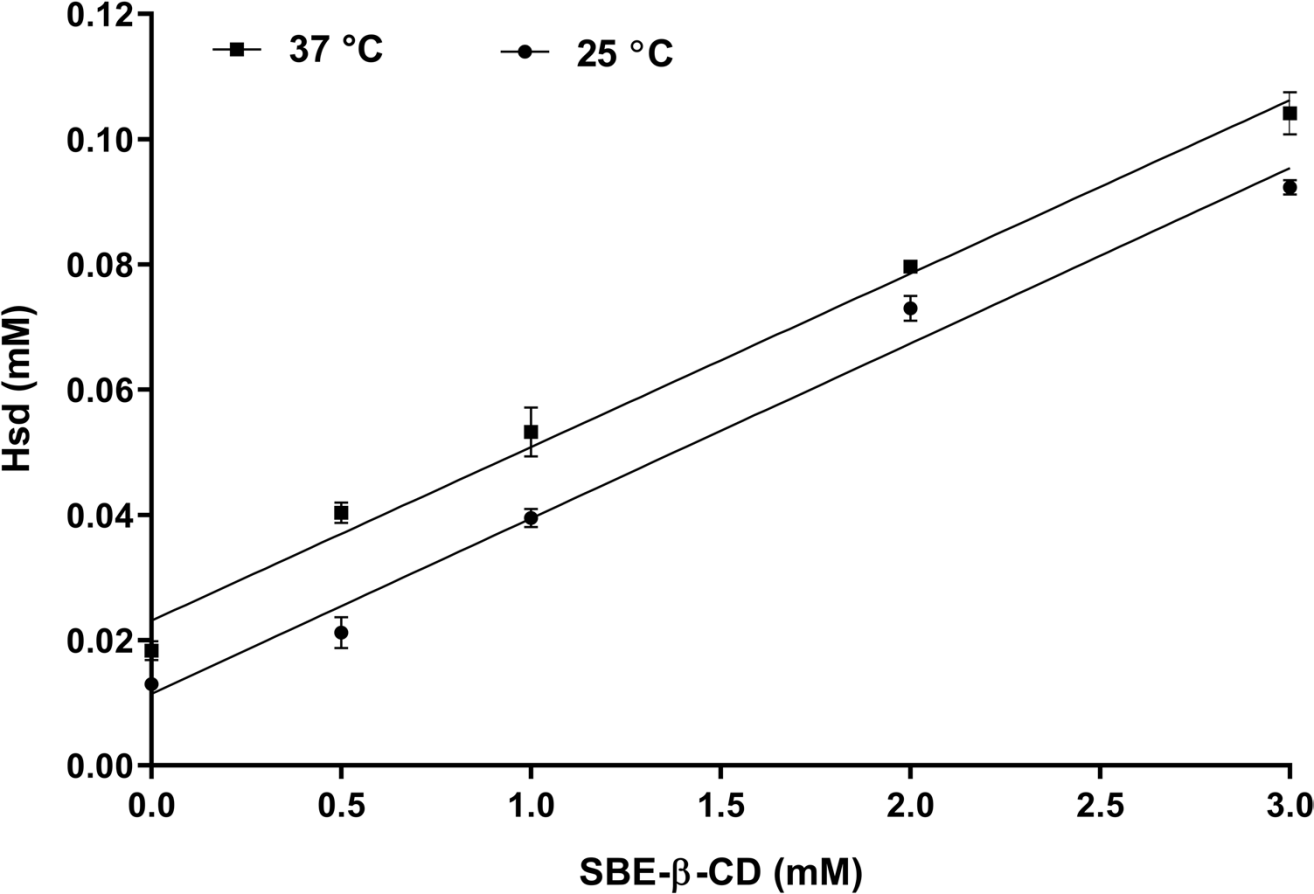
Phase solubility diagram of Hsd in SBE-β-CD. Each point represents the mean ± SD (*n* = 3). Abbreviations: Hsd, hesperidin and SBE-β-CD, sulfobutylether-β-cyclodextrin

The phase solubility diagram of Hsd in SBE-β-CD solutions at 25 ± 0.5 and 37 ± 0.5 °C is illustrated in Fig. 2. Over the studied concentration range, it could be observed that the solubility of Hsd enhanced linearly with rising the SBE-β-CD concentration. Such a pattern could be specified as AL type based on the theory suggested by Higuchi and Connors [77, 78]. As the slope was less than one (0.02799 and 0.02770 for 25, and 37 °C, respectively), the stoichiometry of the complexation was assumed to be 1:1. The intrinsic solubilities (*S*_0_) of Hsd at 25 and 37 °C were 0.01141 and 0.02313 mM, respectively. Additionally, the stability constant (*K*_1:1_) values were 2524 and 1232 M^−1^, at 25 and 37 °C, respectively. The values of *K*_1:1_ suggested the formation of stable CX as they lay between a range of 200 to 5000 M^−1^ which could improve the solubility, bioavailability, and aqueous stability of poorly soluble drugs. Likewise, the *K*_1:1_ was observed to decline with raising temperature from 25 to 37 °C which indicated that the development of the inclusion complex was a spontaneous exothermic process [79].

#### Percent complexation and process efficiency

The values of %CE and %PE of the CX were 50.53 ± 1.46 and 84.52 ± 3.16 %, respectively. These findings were consistent with other investigators [49]. The values of %CE and %PE are dependent on the complexation time and temperature [80]. Therefore, it could be supposed that the employed complexation procedure was effective and provided a satisfying %PE value (more than 60%) [81].

#### FT-IR

**Fig. 3:**
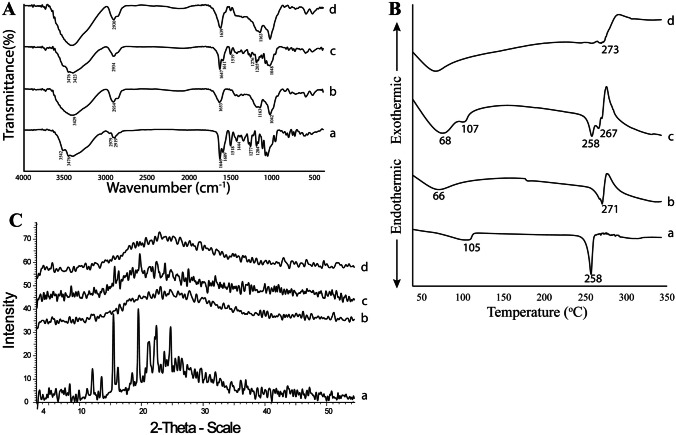
Solid state characterization of Hsd (a), SBE-β-CD (b), Ph_CX_ (c), and CX (d) using FT-IR (**A**), DSC (**B**), and XRD (**C**). Abbreviations: Hsd, Hesperidin; SBE-β-CD, sulfobutylether-β-cyclodextrin; Ph_CX_, physical mixture of hesperidin and sulfobutylether-β-cyclodextrin; and CX, hesperidin: sulfobutylether-β-cyclodextrin complex

Figure 3A illustrates the FT-IR spectra of Hsd, SBE-β-CD, Ph_CX_, and CX. In the spectrogram of Hsd (a), strong bands of OH at 3542 and 3476 cm^−1^, CH (aliphatic) at 2979 and 2919 cm^−1^, C=C (aromatic) at 1609, 1518, and 1444 cm^−1^, C-O at 1277 and 1204 cm^−1^, and C=O at 1646 cm^−1^ could be observed [4, 82]. The SBE-β-CD spectrum (b) exhibited characteristic peaks at 3429 and 2934 cm^−1^ related to the O–H and C–H stretching vibrations, respectively. Besides, the H-O-H stretching peak at 1653 cm^−1^, and a distinctive peak at 1163 cm^−1^ as a consequence of the stretching vibration of C-O-C were noted. Also, the sulfoxide stretching was confirmed by the presence of a strong peak at 1042 cm^−1^ [83, 84]. In the Ph_CX_ spectrum (c), the characteristic absorption peaks of the Hsd at 3476, 1519, 1611, 1276, and 1205 cm^−1^ were maintained. This indicated that Hsd in the Ph_CX_ was not entrapped within the cavity of SBE-β-CD and instead remained uncomplexed. However, sharp peaks of SBE-β-CD dominated in the spectrum of CX (d) with minor shifts of some peaks to lower wave numbers (i.e., 2934 to 2930 cm^−1^; 1653 to 1639 cm^−1^) [43, 85]. The peaks of Hsd were not observed in the CX spectrum which could be ascribed to the inclusion within the cavity of SBE-β-CD [86].

#### DSC

Figure 3B displays the DSC thermograms of Hsd, SBE-β-CD, Ph_CX_, and CX. Hsd (a) showed an endothermic peak at 105 °C due to the removal of the water of crystallization. A second endothermic peak was observed at 258 °C which could be related to the Hsd melting [87]. In the thermogram of SBE-β-CD (b), a broad endothermic peak was found at 66 °C followed by a sharp endothermic peak at 271 °C. The peaks could be attributed to the dehydration of SBE-β-CD followed by its decomposition. The thermogram of Ph_CX_ exhibited just a collection of the endothermic peaks of Hsd and SBE-β-CD (c). The CX thermogram showed a disappearance of Hsd endothermic peaks with slight broadening and shifting of the sharp SBE-β-CD endothermic peak to 273 °C (d). Thus, the formation of the inclusion complex could be confirmed [79].

#### XRD

Figure 3C displays the x-ray diffraction patterns of Hsd, SBE-β-CD, Ph_CX_, and CX. The diffractogram of Hsd (a) displays many characteristic sharp peaks at 12.1, 15.7, 16.5, 19.8, 21.6, 22.5, 23.9, and 25° (2*θ*), which indicated the crystallinity of Hsd [88]. Conversely, the diffractogram of SBE-β-CD (b) exhibited no sharp peaks denoting its amorphous state [89, 90]. The diffraction pattern of Ph_CX_ showed peaks of both Hsd and SBE-β-CD with reduced intensity, revealing a dilution effect due to the presence of two components (c). The diffraction pattern of CX (d) presented an amorphous state with the absence or intensity lessening of the characteristic peaks of Hsd. Such observation deduced the inclusion of Hsd within the cavity of SBE-β-CD associated with diminishing the Hsd crystallinity [91].

#### ^1^H-NMR

**Fig. 4:**
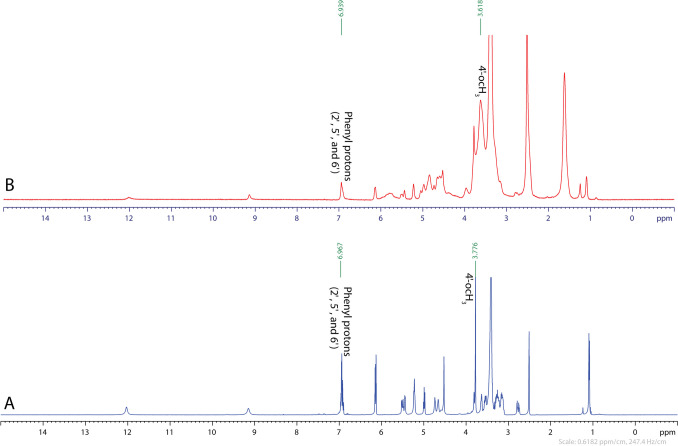
^1^H-NMR spectra of Hsd (**A**) and CX (**B**) in DMSO-*d6*. Abbreviations: Hsd, hesperidin and CX, hesperidin: sulfobutylether-β-cyclodextrin complex

Figure 4 shows the ^1^H-NMR spectra (DMSO-*d6*) of Hsd and CX. The NMR spectroscopy was conducted to figure out the molar ratio as well as the mode of the interaction between Hsd and SBE-β-CD in the inclusion complex. In the chemical structure of Hsd (Fig. 4A), the B-ring is the phenolic electron richer one due to the contribution of the ortho methoxy group [2]. The formation of CX could be predicted via the inclusion of either the methoxyphenyl moiety or glucopyranosyl unit within the core of the SBE-β-CD molecule. But it was questionable that the hydrophilic glucopyranosyl unit of Hsd was hosted within the lipophilic cavity of SBE-β-CD. However, in our state, due to the coexistence of glucopyranosyl units in Hsd and SBE-β-CD, shifting in H3 and H5 peaks of SBE-β-CD was indistinguishable. Instead, a shifting in ^1^H-NMR peaks of methoxy (4′-OCH_3_) and phenyl protons (2′, 5′, and 6′) of B-ring of Hsd from 3.776 to 3.618 *ppm* and from 6.967 to 6.939 *ppm* was observed, respectively (Fig. 4B). Other peaks of A-ring protons as H6 and H8 remained almost unaltered, ensuring that the inclusion phenomenon of Hsd molecule happened via the modulation of the B-ring into the cavity of SBE-β-CD at molar ratio of 1:1 [92].

#### SEM

**Fig. 5:**
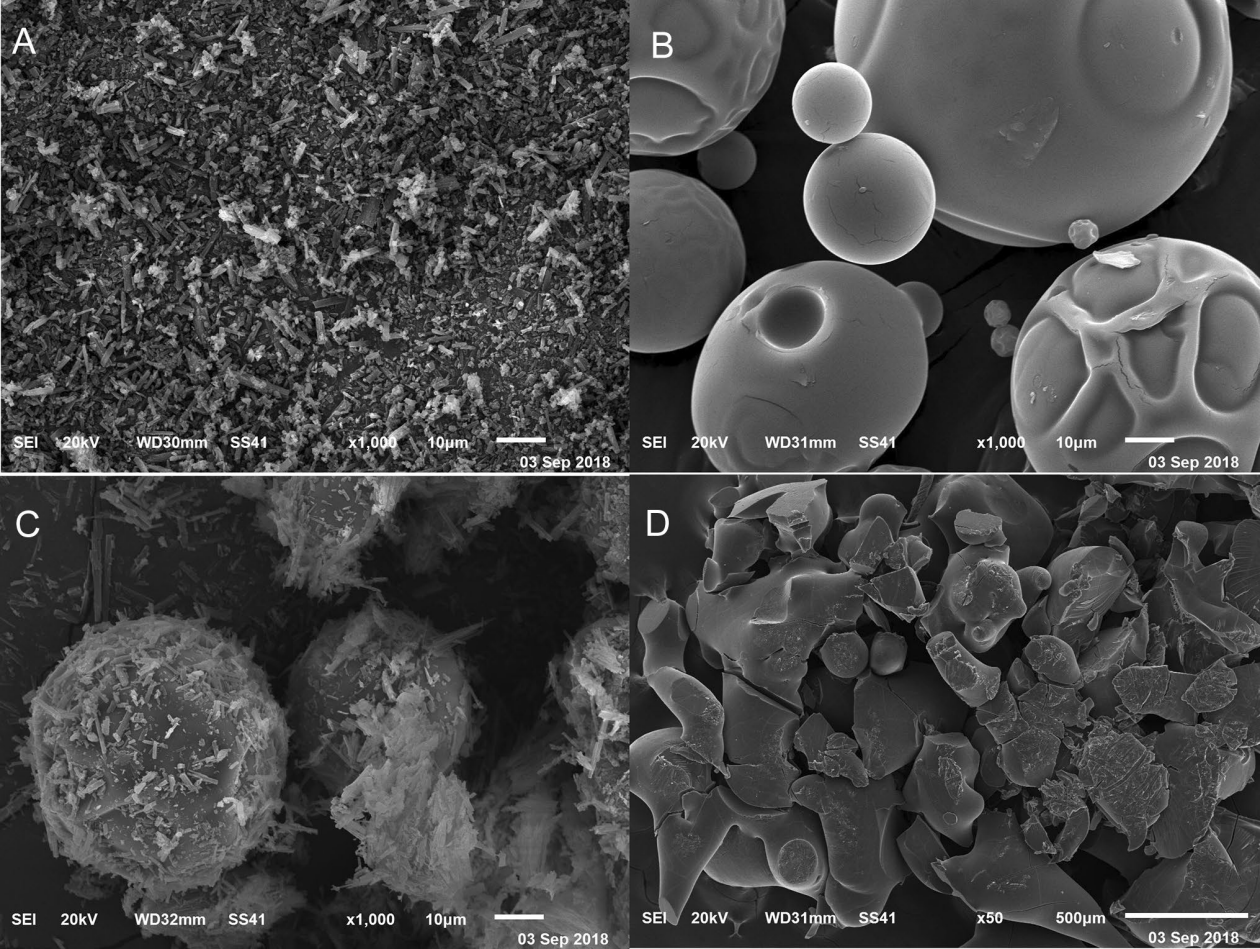
SEM photomicrographs of Hsd (**A**), SBE-β-CD (**B**), Ph_CX_ (**C**), and CX (**D**). Abbreviations: Hsd, hesperidin; SBE-β-CD, sulfobutylether-β-cyclodextrin; Ph_CX_, physical mixture of hesperidin and sulfobutylether-β-cyclodextrin; and CX, hesperidin: sulfobutylether-β-cyclodextrin complex

Figure 5 presents the surface morphology of Hsd, SBE-β-CD, Ph_CX_, and CX. It could be observed that Hsd showed needle-shaped crystals (Fig. 5A) [93, 94]. However, SBE-β-CD exhibited amorphous spherical particles of different sizes (Fig. 5B). The Ph_CX_ morphology demonstrated the Hsd needle crystals attached to the surface of SBE-β-CD particles while both species preserved their authentic morphology (Fig. 5C). Conversely, the surface morphology of CX (Fig. 5D) displayed erratic particles different from host and guest units, confirming the formation of the inclusion complex [85, 95].

### Characterization of Hsd/CD/CS NPs

#### Entrapment efficiency

The EE% of the prepared Hsd/CD/CS NPs is listed in Table 2. It could be perceived that the EE% of Hsd gradually increased from 14.41 ± 7.00% (F5) to 49.7 ± 3.96% (F2) by increasing the SBE-β-CD equivalent amount of CX from 33.5 to 62.5 mg, respectively. Compared to F2, a significant rise (*P* < 0.01) of the EE% to attain a value of 77.46 ± 0.39% was achieved by augmenting the SBE-β-CD amount to 75 mg (F1). This means that EE% was directly proportional to the amount of CX. Such finding could be attributed to the possible increment of the electrostatic interaction between the negatively charged sulfonyl groups of SBE-β-CD and the protonated amino groups of CS at a high concentration of SBE-β-CD resulting in higher EE% (Table 2) [96].

**Table 2:** Characterization of Hsd/CD/CS NPs

**Formulation code**	**Particle size (nm)**	**PDI**	**ZP (mV)**	**EE%**	**Mucin binding efficiency %**
**F1**	455.7 ± 9.04	0.219 ± 0.01	+32.28 ± 1.12	77.46 ± 0.39	48.70 ± 3.54
**F2**	440.7 ± 26.77	0.208 ± 0.02	+34.78 ± 4.49	49.70 ± 3.96	54.87 ± 1.86
**F3**	356.9 ± 12.01	0.208 ± 0.05	+34.40 ± 1.47	32.48 ± 10.04	53.77 ± 8.90
**F4**	447.4 ± 23.47	0.433 ± 0.12	+35.53 ± 0.57	27.41 ± 4.05	55.27 ± 7.17
**F5**	588.7 ± 5.51	0.582 ± 0.16	+42.28 ± 3.58	14.41 ± 7.00	58.50 ± 2.70

Each value represents the mean ± SD (*n* = 3)

*PDI* polydispersity index, *ZP* zeta potential, *EE%* entrapment efficiency percentage

#### Particle size, PDI, and ZP

Table 2 documents the particle size, PDI, and ZP of Hsd/CD/CS NPs. The obtained finding confirmed that the prepared NPs were within the nanometric scale (< 1000 nm) and less than 500 nm except for F5 (588.7 ± 5.51 nm). For oral delivery, the particle size < 500 nm favors enterocytic uptake. Moreover, it has been stated that the polymeric NPs of less than 500 nm can traverse the M cells in the intestine, definitely taken up by the lymphatic system, thus overcoming the first pass hepatic metabolism and increasing the bioavailability [97]. The data clarified an eminent decrement in the particle size of NPs (*P* < 0.0001) from 455.7 ± 9.04 nm (F1) to 356.9 ± 12.01 nm (F3) manifested by reducing the SBE-β-CD amount from 75 to 50 mg. This obvious decrease in the particle size of F3 might be related to the corresponding CS:CX ratio (1:1). Such mass ratio might enhance a further electrostatic interaction resulting in a higher degree of tightness and compaction among the network structure of the Hsd/CD/CS NPs. After that, a further increase of the CS ratio (CS:CX 1:0.67; F5) reflected a significant increment in the particle size up to 588.7 ± 5.51 nm (F5, *P* < 0.0001) [98]. The highest particle size of F5 could be related to the lowest amount of SBE-β-CD as well as the high viscosity of the system. This would lead to a limited inter and intra-molecular crosslinking between CS and CX with a lot of CS molecules remaining unlinked [99]. The excessive positive charge of the free protonated amino groups of the unlinked CS might result in an electrostatic repulsion between the chain of CS followed by swelling, enlargement, and growth of the formed NPs (F5).

The homogeneity of particle size is expressed as PDI. In our study, the prepared Hsd/Cd/CS NPs displayed a narrow size distribution (PDI values less than 0.5) for F1, F2, F3, and F4 except for F5 (0.582 ± 0.16). It is well known that PDI values less than 0.5 indicate the homogenous nature of the NPs dispersion with narrow size distribution [100–103].

As presented in Table 2, the ZP values of the Hsd/CD/CS NPs from F1 to F4 had a positive surface charge with an amplitude ranging from +32.28 ± 1.12 to +35.53 ± 0.57 mV. After that, F5 with the highest CS:CX ratio (1:0.67) demonstrated an abrupt significant increase of the ZP to reach +42.28 ± 3.58 mV (*P* < 0.05), compared to F4. Typically, the NPs with ZP of more than ±30 mV intend to be considered stable, where they generate efficient repulsive forces to secure better physical colloidal stability, while those with ZP of ±15 mV or less are experienced to be unstable with the possibility of the NPs aggregation [104, 105]. The electrical surface charge of NPs is affected by the particle components and dispersion environment. Moreover, the electrostatic repulsion between NPs significantly affects particle stability, mucoadhesive character, and cellular uptake [106, 107]. The positively charged surface of the Hsd/Cd/CS NPs could be ascribed to the protonated amino groups of CS. The highest ZP value of F5 could be related to the abundance of the protonated amino groups at the higher CS concentration, causing sturdy electrostatic repulsion between NPs. These data are in unison with earlier investigations [108].

#### Mucoadhesive strength

The mucin-binding efficiencies of the Hsd/Cd/CS NPs are listed in Table 2. It could be observed that the NPs displayed a synchronized increase in the mucin-binding efficiency with the values of ZP. Accordingly, F5 with the highest ZP value of +42.28 ± 3.58 mV possessed the maximum mucin-binding efficiency of 58.50 ± 2.70%. However, other formulations exhibited comparable mucoadhesive activities with values not less than 48.70 ± 3.54% (F1). Simply, the mucoadhesive efficiency could be related to the CS ratio. As the CS amount increased, both the ZP and the mucin-binding efficiency increased. The mucoadhesive nature of CS is related to its cationic character. On the other hand, the acidic sialic and sulfonic moieties of mucin are responsible for its anionic functionalities. The ionic interaction between the cationic groups of CS and anionic acids of the mucin secures the mucoadhesive activity of CS [109, 110]. For oral delivery, this interaction could enhance the gastrointestinal residence time and the enterocyte uptake of the NPs which is crucial for efficient mucosal surrender of therapeutics [111]. Hence, the mucoadhesive property of CS NPs improves both the absorption and the bioavailability of the drug due to prolonged contact with the mucosal layer accompanied by the high surface-to-volume ratio of the NPs [112].

To sum up the above results, F1 presented the maximum EE% of 77.46 ± 0.39%, and particle nano size of 455.7 ± 9.04 nm with narrow distribution (PDI = 0.219 ± 0.01). Moreover, a surface charge of +32.28 ± 1.12 mV resulted in appropriate mucoadhesive strength of 48.70 ± 3.54%. Hence, F1 was subjected to the forthcoming evaluations.

### Characterization of the optimal formula (F1)

#### Transmission electron microscopy (TEM)

**Fig. 6:**
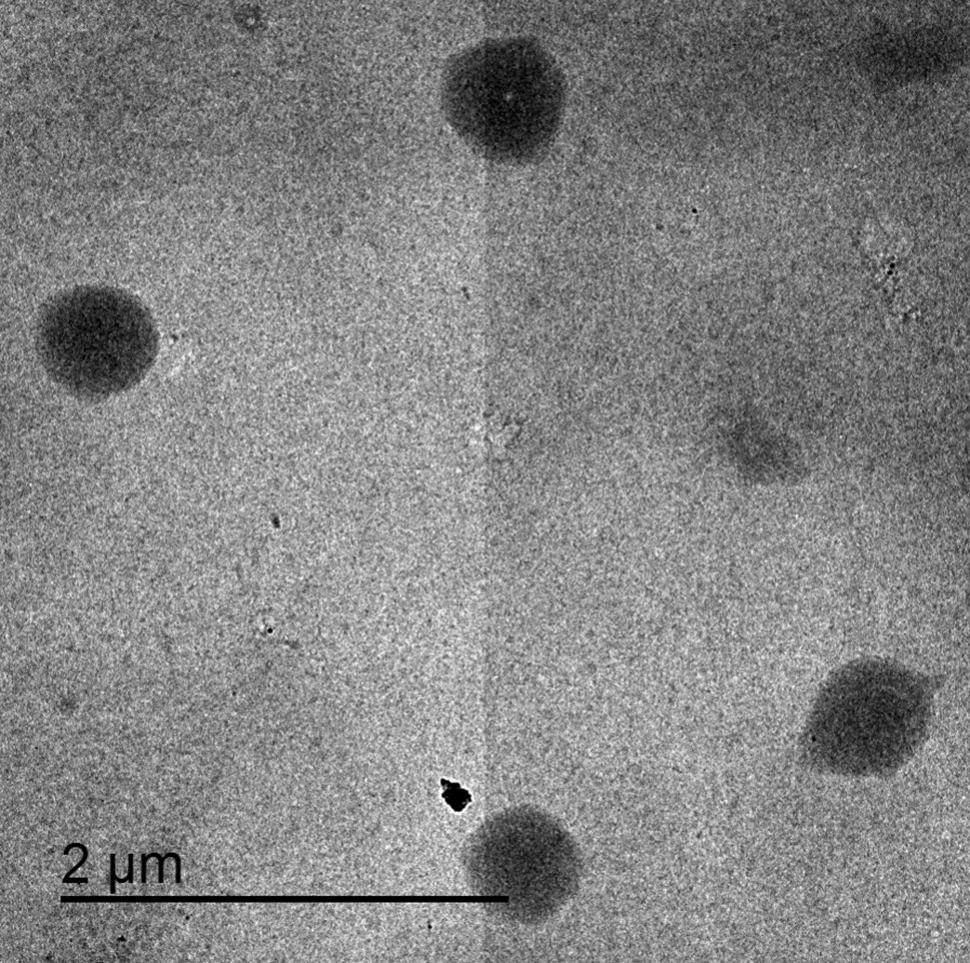
TEM photomicrograph of F1. Abbreviation: F1, Hsd/CD/CS NPs optimal formula

Figure 6 shows the TEM micrograph of F1. The TEM affirmed that F1 had aggregate-free spherical-shaped particles. Including the surface and size attributes of NPs, the shape has a magnificent effect on the biological system interaction, enterocytic uptake, and bio-distribution [113]. It had been reported that the spherical-shaped NPs have a higher capacity for cellular internalization in comparison with their rod equivalents. Contrary to the rod NPs, the spherical ones have not extremely affected by the cytoskeleton and are eventually uptaken into cells at a faster rate [114]. Furthermore, the size analysis revealed that the particle size was consistent with that measured by the DLS technique [115, 116].

#### SEM

**Fig. 7:**
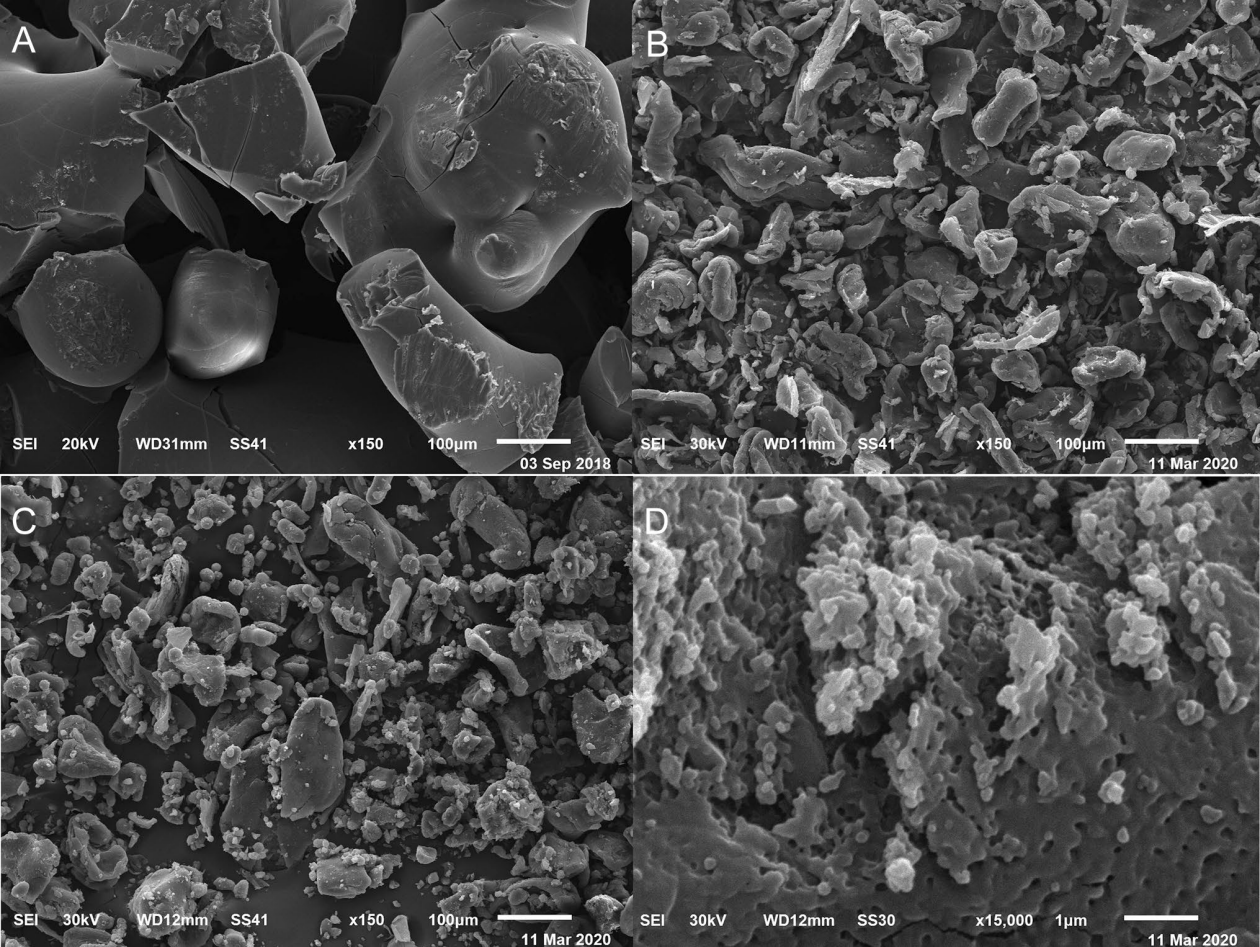
SEM photomicrographs of CX (**A**), CS (**B**), Ph_NPs_ (**C**), and F1 (**D**). Abbreviations: CX, hesperidin: sulfobutylether-β-cyclodextrin complex; CS, chitosan; Ph_NPs_, physical mixture of the complex and chitosan; and F1, Hsd/CD/CS NPs optimal formula

Figure 7 illustrates the SEM images of CX, CS, Ph_NPs_, and F1. The erratic particle morphology of CX was clear (Fig. 7A) [79]. The surface morphology of CS (Fig. 7B) displayed irregular block structures and sometimes elongated sticks [117, 118]. The surface morphology of Ph_NPs_ showed a collection of the individual components (Fig. 7C) [83, 119]. Finally, F1 (Fig. 7D) displayed spherical regular particles embedded within a porous matrix [120, 121].

#### FT-IR

**Fig. 8:**
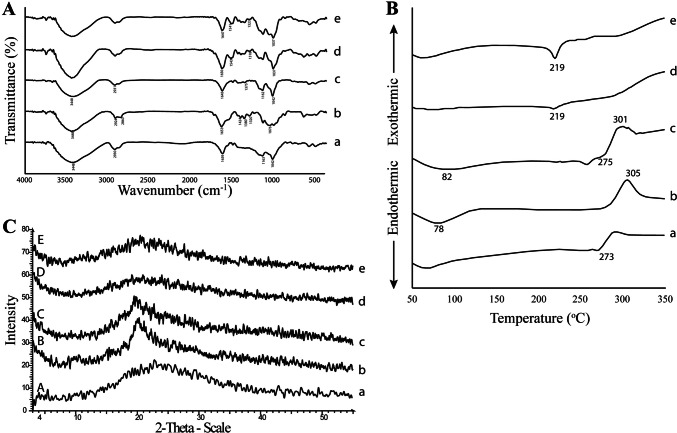
Solid state characterization of CX (a), CS (b), Ph_NPs_ (c), PLF1 (d), and F1 (e) using FT-IR (**A**), DSC (**B**), and XRD (**C**). Abbreviations: CX, hesperidin: sulfobutylether-β-cyclodextrin complex; CS, chitosan, Ph_NPs_, physical mixture of the complex and chitosan; PLF1, plain CD/CS NPs optimal formula; and F1, Hsd/CD/CS NPs optimal formula

The FT-IR spectra of F1, and its components as well as PLF1 are presented in Fig. 8A. The CX spectrum (a) was previously discussed under the “FT-IR” section. CS spectrum (b) showed a strong peak at 3448 cm^−1^ assigned to the stretching of the O-H bond and N-H stretching of primary amines as well as the intermolecular H-bonding. A peak at 1076 cm^−1^ and another sharp one at 1424 cm^−1^ could be related to the symmetrical stretching of C-O-C and C-N, respectively. The mode of vibration at 2860 and 2924 cm^−1^ was related to C-H stretching [122]. Also, a strong sharp peak of amide I at 1653 cm^−1^ and amide III at 1320 cm^−1^ were clear. Furthermore, the presence of the CH_2_-OH functional group was evident and figured out as a sharp peak at 1380 cm^−1^ [123]. The spectrum of Ph_NPs_ displayed a superposition of the CX and CS spectra (c) [124]. By synchronizing the PLF1 and F1 spectra with those of CS and CX (d and e, respectively), spectral changes were experienced indicating an electrostatic interaction between SBE-β-CD anionic sulfo-butyl groups and CS cationic amine groups. So, the amide I peak shifted from 1653 to 1650 and 1647 cm^−1^ and the amide III peak shifted from 1320 to 1318 and 1323 cm^−1^ for PLF1 and F1, respectively [125]. Also, the peak of amide II appeared at 1543 and 1541 cm^−1^ for PLF1 and F1, respectively [126]. Furthermore, the sulfoxide stretching of 1042 cm^−1^ shifted to 1037 cm^−1^ in both spectra of PLF1 and F1 [127]. Such findings could prove the existence of electrostatic interaction between CS and CX. Subsequently, the formation of the CS NPs could be established.

#### DSC

Figure 8B depicts the DSC thermograms of CX, CS, Ph_NPs_, PLF1, and F1. The thermogram of CX (a) was discussed under the “DSC” section, where the endothermal peaks of Hsd disappeared. The CS thermogram (b) showed an endothermic peak at 78 °C referring to the loss of the bound water. Also, another exothermic characteristic peak appeared at 305 °C as a result of the polymer degradation [128, 129]. The Ph_NPs_ thermogram exhibited characteristic peaks of the individual components (c). The PLF1 and F1 thermograms (d and e, respectively) showed that the decomposition peak of SBE-β-CD shifted from 273 to 219 °C confirming the ionic interactions between CS and SBE-β-CD which paved the formation of the NPs [130]. Moreover, the endothermic peak of CS at 78 °C was observed in the range of 60–100 °C indicating that the heat generation during the cross-linking between SBE-β-CD and CS might alter the enthalpy of the NPs [131].

#### XRD

Figure 8C illustrates the XRD diffractograms of CX, CS, Ph_NPs_, PLF1, and F1. The diffraction pattern of CX showed its amorphous nature which was previously described under the “XRD” section. (a). Likewise, CS (b) showed one broad characteristic peak at 20° of 2*θ* indicating its amorphousness [126]. The diffractogram of Ph_NPs_ depicted the overlay of the single components (c) [130]. Broadness was observed in both PLF1 and F1 diffractograms with diminishing peak intensities (d and e, respectively). This could be ascribed to intermolecular hydrogen bonding. Moreover, the complexation of Hsd was responsible for the reduction in its peak intensity which in turn contributes to the amorphousness of the CS NPs [132, 133]. Such observation indicated that the presence of the complexed Hsd did not disturb the nanostructured architecture of the NPs [134].

### In vitro release study

**Fig. 9:**
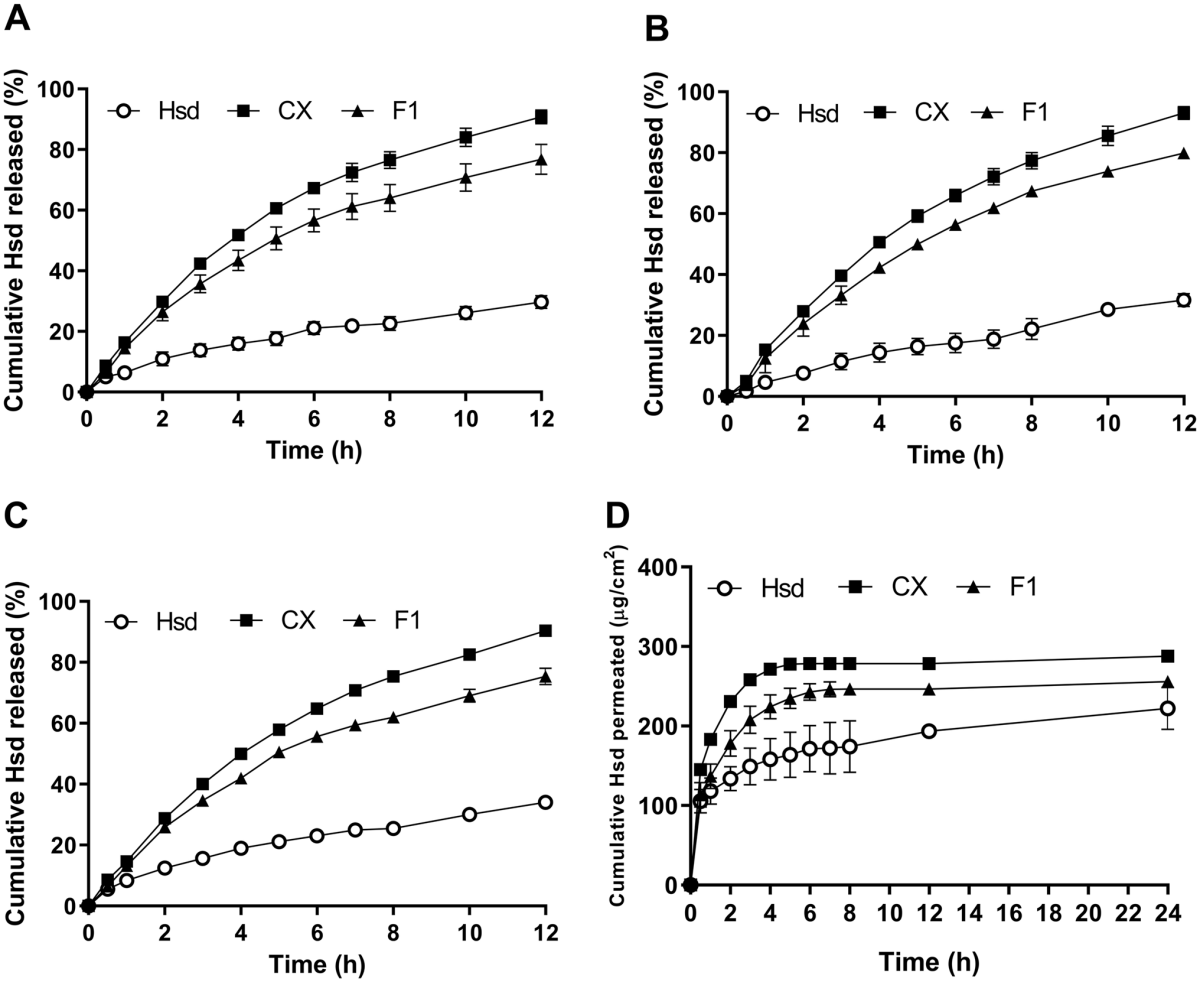
In vitro release study at pH 1.2 (**A**), 6.8 (**B**), 7.4 (**C**), and the ex vivo intestinal permeation study at pH 7.4 (**D**) of Hsd, CX, and F1. Each point represents the mean ± SD (*n* = 3). Abbreviations: Hsd, hesperidin; CX, hesperidin: sulfobutylether-β-cyclodextrin complex; and F1, Hsd/CD/CS NPs optimal formula

The in vitro release patterns of Hsd from CX and F1 in comparison with its diffusion from the aqueous dispersion are illustrated in Fig. 9A–C. Methanolic solutions (40% v/v) at pH 1.2, 6.8, and 7.4 were used as release media to mimic the gastric, intestinal, and physiological environment, respectively. Starting with the gastric pH of 1.2 (Fig. 9A), the free Hsd showed a slow-release pattern to attain only 29.70 ± 2.10% after 12 h. On the other hand, the CX displayed an obvious increase in the release rate of Hsd to reach 90.77 ± 2.25% after 12 h. F1 exhibited an intermediate release percent of 76.77 ± 4.97%. In the intestinal pH of 6.8 (Fig. 9B), the free Hsd and CX offered a percent release of 31.60 ± 2.08 and 93.00 ± 2.12% after 12 h, respectively. While F1 modulated the release of the CX to a slower rate getting 79.90 ± 1.56% within 12 h. At the physiological pH (Fig. 9C), the free Hsd, CX, and F1 exhibited a similar release manner as the above-mentioned ones with a percent of 34.00 ± 1.13, 90.40 ± 1.56, and 75.40 ± 2.69%, respectively. The slowest release rate of the Hsd  dispersion could be attributed to its poor aqueous solubility [135–137]. The complexation of Hsd raised the Hsd solubility (“Phase solubility” section) and diminished the Hsd crystallinity as proved under the CX detailed characterization (“Percent complexation and process efficiency”, “FT-IR”, “DSC”, “XRD”, “1H-NMR” and “SEM” sections). Hence, CX had a much higher percentage of Hsd release at the studied pH release media [138, 139]. The sustained release of F1 could be referred to that the complexed Hsd had to first depart the cavity of SBE-β-CD (complex dissociation) then, cross the network configuration of the CS NPs, and finally diffuse to the release media [140].

### Kinetic analysis

Table 3 displays the coefficients of determination of Hsd, CX, and F1 at different pH release media. It could be noted that the Hsd release from its dispersion followed the Higuchi model at both the gastric and the physiological simulating conditions, while the first-order kinetic model fitted its release in the intestinal pH with *R*^2^ values of 0.994, 0.9961, and 0.9884, respectively. The Hsd release from CX and F1 in the gastric, intestinal, and physiological pH was found to follow first-order kinetics with *R*^2^ values exceeding 0.9845 (Table 3). For more exploration of the release kinetics, the Korsmeyer-Peppas equation was employed. The values of *n* are listed in Table 3. It could be noticed that Hsd showed anomalous (non-Fickian) behavior combining both the molecular diffusion and the particle erosion where the n exponents were in the range of 0.5624–0.8692. Also, it was the case for the CX and F1 at pH 1.2 and 7.4. In the intestinal pH (6.8), CX and F1 offered Super case II transport (*n* ˃ 0.89), where the character of dissolution dominated [62].

**Table 3:** Kinetic analysis of the release data of Hsd from aqueous Hsd dispersion, aqueous CX solution, and F1 dispersion at different pH release media

**pH release media**	**Formula**	**Zero order**	**First order**	**Higuchi**	**Korsmeyer-Peppas**		**Main transport mechanism**
		***R***^**2**^			***R***^**2**^	***n***	
**1.2**	**Hsd**	0.9394	0.9602	0.994	0.9936	0.5805	Anomalous (non-Fickian) diffusion
	**CX**	0.9232	0.9962	0.9846	0.9982	0.8498	
	**F1**	0.923	0.9932	0.9856	0.988	0.8645	
**6.8**	**Hsd**	0.9817	0.9884	0.9591	0.9846	0.8692	Anomalous (non-Fickian) diffusion
	**CX**	0.938	0.9845	0.9808	0.9766	1.039	Super case II transport
	**F1**	0.9423	0.9989	0.9789	0.9733	1.058	
**7.4**	**Hsd**	0.9373	0.9622	0.9961	0.998	0.5624	Anomalous (non-Fickian) diffusion
	**CX**	0.9353	0.9933	0.9846	0.9978	0.8485	
	**F1**	0.9234	0.9907	0.9845	0.9899	0.8429	

*Hsd* hesperidin, *CX* Hsd/SBE-β-CD complex, *F1* Hsd/CD/CS NPs optimal formula, *R*^*2*^ coefficient of determination, and *n* diffusion exponent

### Ex vivo intestinal permeation

The cumulative Hsd amount permeated (µg/cm^2^) across the goat intestine from Hsd dispersion, CX, and F1 is depicted in Fig. 9D. Also, Table 4 displayed the permeation parameters of the studied formulations. The Hsd dispersion showed that an amount of 222.3 ± 26.52 µg/cm^2^ of Hsd permeated after 24 h, while CX and F1 depicted cumulative permeated amounts of 287.9 ± 7.29 and 255.6 ± 6.69 µg/cm^2^, respectively. The CX depicted a significant increase of *J*_ss_ (44.95 ± 0.406 µg/cm^2^.h) and *K*_p_ (0.193 ± 0.002 cm/h) (*P* < 0.01), in comparison to those of Hsd dispersion (20.29 ± 4.657 µg/cm^2^.h and 0.087 ± 0.019 cm/h, respectively). The incorporation of CX in F1 demonstrated a significant reduction of the *J*_ss_ and *K*_p_ values to reach 28.13 ± 0.241 µg/cm^2^.h and 0.121 ± 0.001 cm/h, respectively (*P* < 0.05). The intestinal permeability of the studied formulation could be arranged as the following: CX > F1 > Hsd. Hsd has been reported to have a weak membrane permeability and consequently, its absorption mainly occurs via the paracellular pathway. This means that the tight junctions of enterocytes might hinder Hsd oral absorption and bioavailability [141, 142]. Due to the poor aqueous solubility of Hsd, increasing its solubility by the complexation could raise its intestinal permeability [143]. Moreover, SBE-β-CD can inhibit P-gp ATPase action, which in sequence increases intestinal permeability [144]. CS is assumed to improve paracellular transport ensuing a structural reorganization of tight junction-associated proteins and then enhancement of intestinal permeation. Moreover, CS is characterized by dedicated mucoadhesive property due to its positive charge which augments the interaction of CS NPs with the intestinal mucosa [145, 146]. This condition might permit an intimate contact, and extended residence time of F1 to prolong its intestinal permeation [147].

**Table 4:** Cumulative permeated amount, flux, and permeability coefficient of Hsd, CX, and F1

**Formula**	**Cumulative permeated amount (µg/cm**^**2**^**)**	***J***_**ss**_ **(µg/cm**^**2**^**.h)**	***K***_**p **_**(cm/h)**
**Hsd**	222.3 ± 26.52	20.29 ± 4 .657	0.087 ± 0.019
**CX**	287.9 ± 7.29^***^	44.95 ± 0.406^***^	0.193 ± 0.002^**^
**F1**	255.6 ± 6.69^*^	28.13 ± 0.241^*^	0.121 ± 0.001

Each value represents the mean ± SD (*n* = 3)

*Hsd* hesperidin, *CX* Hsd/SBE-β-CD complex, *F1* Hsd/CD/CS NPs optimal formula, *J*_*ss*_ flux, and *K*_*p*_ permeability coefficient

*P < 0.05; **P < 0.01; ***P < 0.0001 versus the corresponding value of Hsd

### Stability study

Table 5 documents the stability of F1 designated as particle size, PDI, ZP, and DR%. Over the storage period, it was observed that the storage temperatures affected the change rate of the measured parameters. At the ambient temperature, the initial particle size and PDI started to increase significantly during the storage reaching 540.9 ± 4.86 nm and 0.712 ± 0.006, respectively, after 6 months (*P* < 0.0001). Additionally, at the end of the study, the values of ZP and DR% declined significantly to become + 25.60 ± 0.10 mV and 90.70 ± 1.30%, respectively (*P* < 0.0001). Alternatively, the storage of F1 at 4 ± 1 °C for 6 months resulted in a particle size of 464.8 ± 2.64 nm and a ZP of + 31.27 ± 0.25 mV which were not significantly different from those of the corresponding ones at zero time. The values of PDI and DR% did not exceed 0.156 ± 0.003 and 95.82 ± 1.86%, respectively. These data emphasized the superlative stability of F1 at refrigerated temperature, thereby preserving its physicochemical efficacy for long-term storage [70, 145, 148].

**Table 5:** Stability study of F1 at ambient and refrigerated (4 ± 1 °C) temperatures

**Parameter**		**Storage time**			
		**Zero time**	**1 month**	**3 months**	**6 months**
**Ambient temperature**	**Particle size (nm)**	455.7 ± 9.04	472.3 ± 1.65^*^	486.9 ± 5.30^***^	540.9 ± 4.86^***^
	**PDI**	0.219 ± 0.005	0.121 ± 0.01^***^	0.539 ± 0.019^***^	0.712 ± 0.006^***^
	**ZP (mV)**	+32.28 ± 1.12	+30.33 ± 0.38^*^	+27.39 ± 0.19^***^	+25.6 ± 0.10^***^
	**DR%**	100.00 ± 0.00	95.05 ± 1.38^**^	92.84 ± 0.88^***^	90.7 ± 1.30^***^
**Refrigerator temperature (4 ± 1 °C) **	**Particle size (nm)**	455.7 ± 9.04	459.1 ± 7.41	460.6 ± 1.04	464.8 ± 2.64^a^
	**PDI**	0.219 ± 0.005	0.128 ± 0.015^***^	0.133 ± 0.003^***^	0.156 ± 0.003^a***^
	**ZP (mV)**	+32.28 ± 1.12	+32.47 ± 1.06	+31.76 ± 0.52	+31.27 ± 0.25^a^
	**DR%**	100 ± 0.00	98.88 ± 0.82	98.43 ± 0.43	95.82 ± 1.86^a**^

Each value represents the mean ± SD (*n* = 3)

*PDI* polydispersity index, *ZP* zeta potential, *DR%* drug retention percentage, *F1* Hsd/CD/CS NPs optimal formula

*P < 0.05; **P < 0.001; ***P < 0.0001 versus the corresponding value at zero time

^a^*P* < 0.0001 versus the corresponding value at ambient temperature

### Cytotoxicity assay

**Fig. 10:**
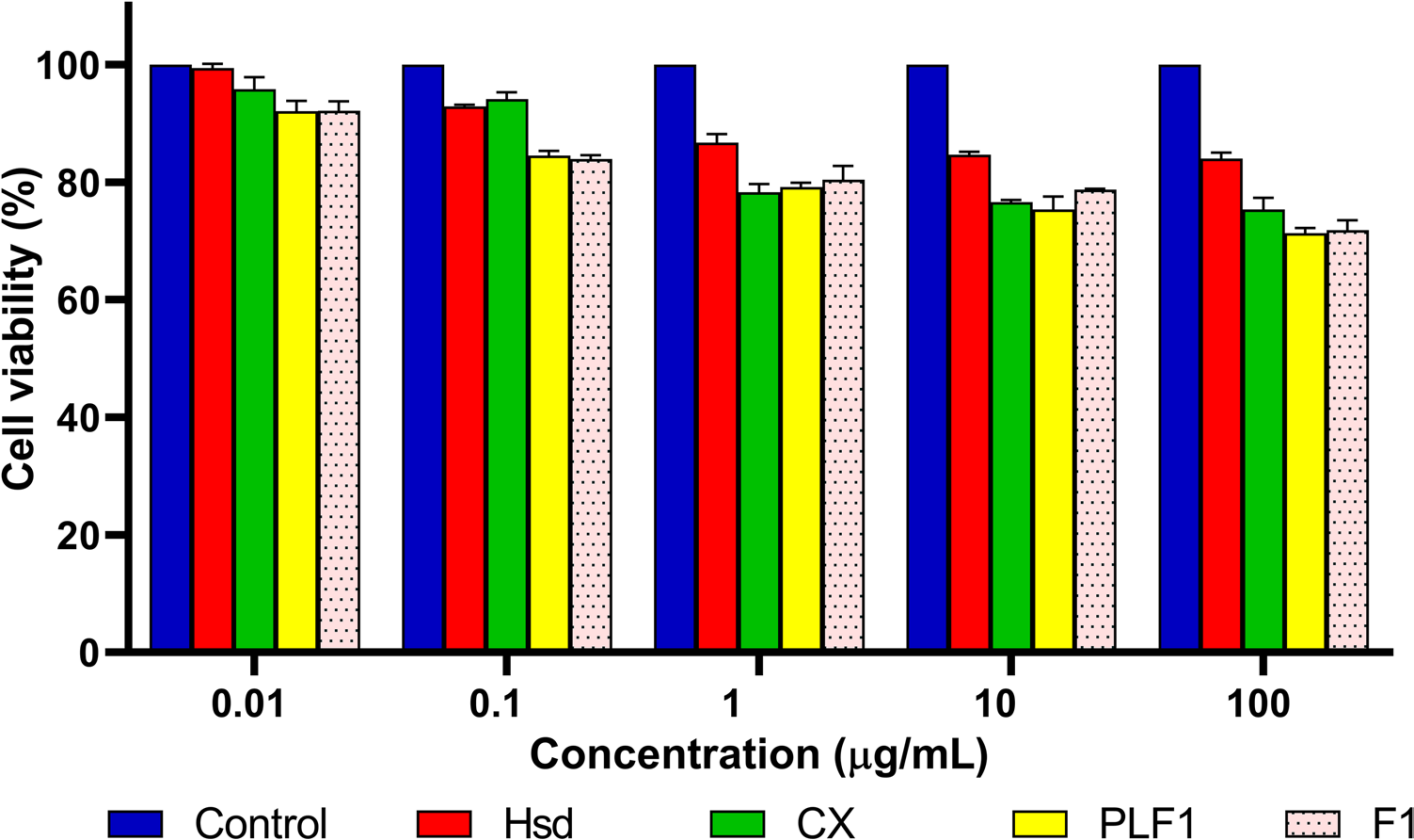
The percent cell viability of OEC at different concentrations of Hsd, CX, PLF1, and F1. Each point represents the mean ± SD (*n* = 3). Abbreviations: OEC, the normal oral epithelial cell line; Hsd, hesperidin; CX, hesperidin: sulfobutylether-β-cyclodextrin complex; PLF1, Plain CD/CS NPs optimal formula; and F1, Hsd/CD/CS NPs optimal formula

Figure 10 displays the cell viability of OEC at different concentrations of Hsd, CX, PLF1, and F1 using the WST-1 assay. In literature, Hsd has shown possible cytotoxic activities on several cancer cell lines [149–151]. Consequently, to eliminate the anticancer activity of Hsd, it was necessary to investigate the cytotoxicity using a normal cell line representing the oral delivery pathway (oral epithelial cell line; OEC). Referring to the results, it could be observed that the studied formulations offered IC_50_ of more than 100 µg/mL assuring their safety on the OEC. Hsd and CX at the concentration of 100 µg/mL showed cell viabilities of 84.00 ± 1.06 and 75.33 ± 2.01%, respectively. Such a decrement (*P* < 0.0001) in the viability by complexation could be ascribed to the improved solubility of the complexed Hsd which in turn might augment more cellular uptake and internalization. Also, PLF1 and F1 at a concentration of 100 µg/mL preserved comparable cell viability values of 71.29 ± 0.89 and 71.86 ± 1.65%, respectively. However, the cell viability > 70% indicated that the tested formulations were well tolerated. The chief components of F1 were SBE-β-CD and CS which exhibited safety and wide applicability in the drug delivery systems. These results agreed with earlier studies that reported the safety of SBE-β-CD even after its complexation with drugs [144, 152–155]. It is well known that CS is an extensively used biocompatible polymer due to its non-toxic and biodegradable nature [156, 157]. Hence, the obtained finding documented the biocompatibility of F1 over the tested concentration range.

### In vivo evaluation of the pharmacodynamic hypoglycemic effect

**Fig. 11:**
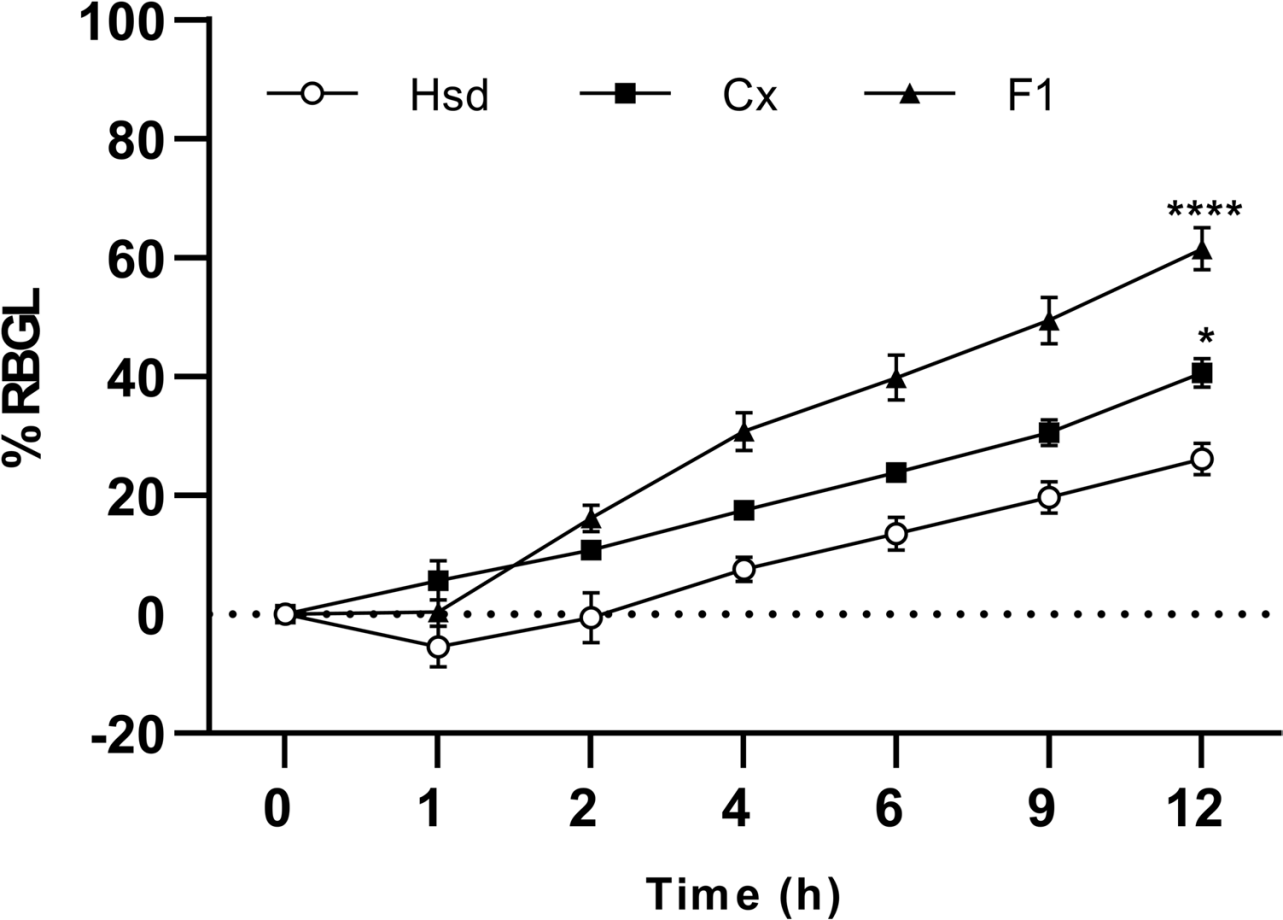
The hypoglycemic patterns of Hsd, CX, and F1 expressed as %RBGL. Each point represents the mean ± SEM (*n* = 3). Statistical significances of rat groups are indicated as *P < 0.05 and ****P < 0.0001compared to Hsd. Abbreviations: %RBGL, the percent reduction in the blood glucose level; Hsd, hesperidin; CX, hesperidin: sulfobutylether-β-cyclodextrin complex; and F1, Hsd/CD/CS NPs optimal formula

The mean %RBGL after the oral administration of Hsd, CX, and F1 was statistically analyzed, and the resultant data is demonstrated in Fig. 11. Throughout the experiment, Hsd exhibited the lowest hypoglycemic activity compared to CX and F1. After 12 h of the oral administration, Hsd showed a %RBGL of 26.11 ± 2.66% while CX revealed a significant %RBGL (*P* < 0.05) of 40.60 ± 2.44%. The oral administration of F1 experienced a delayed reduction of the blood glucose level up to 1 h. Then, a faster reduction rate of the blood glucose level was noted to accomplish a significantly higher %RBGL of 61.43 ± 3.54% (*P* < 0.0001). So, the biological hypoglycemic activity of the investigated formulae can be arranged as the following: F1 > CX > Hsd.

Hyperglycemia may be imputed to reduced hepatic glycogen synthesis together with augmented hepatic glucose production, which may be the reason for reduced glucokinase activities and raised phosphoenolpyruvate carboxykinase and glucose-6-phosphatase activities. The hypoglycemic effect of Hsd could be attributed to the induction of the glucokinase action however declining the phosphoenolpyruvate carboxykinase and glucose-6-phosphatase actions. Moreover, Hsd could ameliorate glucose intolerance and insulin resistance [10, 13]. The solubility of drugs is a key element for their oral absorption. The poor solubility and the minimal permeability of Hsd were early proven and discussed in our study (“Ex vivo intestinal permeation” section). Consequently, the oral administration of Hsd might produce the lowest absorption and the minimal hypoglycemic effect in this study.

Enhancing the solubility of Hsd by complexation with SBE-β-CD improved its dissolution rate and solubility and subsequently could potentiate the oral absorption and pharmacodynamic hypoglycemic effect. Moreover, SBE-β-CD was reported to lessen the viscosity of the intestinal mucus layer and mucosal tissue components (i.e., cholesterol and phospholipids), thereby boosting the permeability at the absorption site [158]. Thus, CX manifested an enhanced hypoglycemic activity over the free Hsd.

It was evident that the hypoglycemic pattern of the orally administered F1 was affected by the physicochemical properties of the CS NPs. The time required for the interaction between the positively charged NPs of F1 (ZP of +32.28 ± 1.12 mV), and the mucosal surface of the gastrointestinal tract might be responsible for this lag time (1 h). Interestingly, studies reported that CS could inhibit hyperglycemia by lessening glucose intestinal digestion, upregulating expression for glucose transporters, and enhancing the cellular uptake of glucose. CS could improve intestinal permeation by its mucoadhesive property and enhanced paracellular pathways [159–161]. Such findings explain the synergistic hypoglycemic effect exerted by F1 that significantly decreased blood glucose levels by the end of the experiment.

## Conclusion

In the present study, the solubility of Hsd was significantly enhanced by complexation with SBE-β-CD. Orally nanoparticulate drug delivery systems (Hsd/CD/CS NPs) were successfully prepared by the ionic gelation of CS and SBE-β-CD. Based on a detailed evaluation of the prepared NPs, F1 was the optimal formula possessing the proper particle size, ZP, EE%, and mucoadhesive characteristics. Further examination of F1 revealed transitional release and intestinal permeation compared to Hsd and CX. Also, F1 had not demonstrated any signs of cytotoxicity over the tested concentrations employing normal OEC. The hypoglycemic effect of F1 was found to be worthwhile and considered as a prospective product of unique combined biological activities of the complexed Hsd and CS.

## Data Availability

The datasets generated during and/or analyzed during the current study are available from the corresponding author upon reasonable request.
